# Systematic Documentation of State Variation in Medicaid Home- and Community-based Services: The Medicaid Home and Community-based Services Dataset Initiative

**DOI:** 10.1093/geroni/igaf044

**Published:** 2025-05-06

**Authors:** Katherine E M Miller, Elise M Parrish, Johanna Thunell

**Affiliations:** Department of Health Policy and Management, Bloomberg School of Public Health, Johns Hopkins University, Baltimore, Maryland, USA; Roger and Flo Lipitz Center to Advance Policy in Aging and Disability, Bloomberg School of Public Health, Johns Hopkins University, Baltimore, Maryland, USA; Department of Sociology, University of Pennsylvania, Philadelphia, Pennsylvania, USA; Leonard Davis Institute of Health Economics, University of Pennsylvania, Philadelphia, Pennsylvania, USA; Sol Price School of Public Policy, University of Southern California, Los Angeles, California, USA; Schaeffer Center for Health Policy and Economics, University of Southern California, Los Angeles, California, USA

**Keywords:** Caregivers, Home-based care, Policy

## Abstract

**Background and Objectives:**

In the United States, Medicaid plays a prominent and growing role as a primary payer for home- and community-based services (HCBS) to help adults remain living independently in the community. While Medicaid HCBS programs vary significantly across states, limited historical, systematic data about Medicaid HCBS program components exist. We presented a systematic, reproducible approach to capture comprehensive characteristics of Medicaid HCBS waivers.

**Research Design and Methods:**

We used current and historical documentation of Medicaid 1915(c) waivers serving adults ages 65 or older from 2010 to 2019. We described waiver services available over time, specifically respite, transport, meals/dietary/nutrition services, caregiver training, and payments to family members for personal care services. We extracted data from waiver documents using HTML parsing.

**Results:**

We extracted data systematically from 419 of 431 waiver documents (97%) across 46 states. During a manual quality control review of data extracted, 9% of waiver documents required any manual corrections, with only 4% requiring significant corrections impacting analysis (eg, missing services). We observed that the percentage of waivers offering each service increased over time for most services except caregiver training, which decreased.

**Discussion and Implications:**

This study fills a critical gap in data availability by demonstrating a systematic approach by which researchers can construct a historical, waiver-level database of Medicaid HCBS waiver characteristics.

**Translational Significance**

Medicaid is the largest payer of long-term care in the United States. Yet, Medicaid home- and community-based services (HCBS) vary significantly across states and limited historical, systematic data about Medicaid HCBS program components exists which impedes rigorous evaluation of the effectiveness of different state approaches. We provided a methodology to contribute an important piece of the puzzle to better understand the HCBS services available through Medicaid. This work lays the foundation for future work to conduct a rigorous evaluation of the comparative effectiveness of Medicaid HCBS on health outcomes, healthcare use, and costs for older adults with disability.

In the United States, systems for delivering long-term care have shifted away from care provided in institutions toward care in the community, reflecting both preferences to age in place and policy, such as the 1999 Olmstead Supreme Court decision supporting independent community living when possible. As a growing proportion of older adults age in place, demand for medical and social services provided in home- and community-based settings to age in place and maintain quality of life is expected to increase. Without a universal long-term care insurance program, most home- and community-based services (HCBS) are paid for out-of-pocket or by Medicaid, a jointly funded federal and state program that covers over half of the $286.5 billion spent on HCBS in the United States (1). Federal law requires state Medicaid programs to cover nursing home care, but states can offer HCBS through various waivers (eg, 1915(c), 1115), provided costs do not exceed institutional care costs (2–4). Through 1915(c) waivers specifically, states can experiment with HCBS program characteristics, such as expanding services offered and establishing different financial eligibility criteria and need criteria for institutional placement (3,5). Nearly all states have at least one 1915(c) waiver, with over 250 waivers currently active; 47 states have a total of 65 active 1115 waivers (6,7). Policy changes focusing on HCBS have primarily focused on 1915(c) waivers, which comprise 50% of Medicaid HCBS spending when examining spending across program authorities (1,5,8). Despite their prominent and growing role, Medicaid HCBS programs vary significantly across states. Yet, limited historical, systematic data about Medicaid HCBS program components exist.

The lack of data about Medicaid HCBS waiver characteristics has impeded rigorous evaluation of the effectiveness of these waivers on health outcomes, healthcare use, and costs for Medicaid HCBS recipients. Existing research examining the effects of HCBS waivers is largely constrained to analyses of single states/programs that often lack a comparison group similar to Medicaid HCBS recipients—a key element to understanding program effects (9–12). Alternatively, some researchers aggregated waiver participants nationally and compared them to nonwaiver participants (which assumes equivalent impacts of waivers) or used waiver expenditures as a proxy for waiver characteristics, thereby potentially masking heterogeneous effects of waiver characteristics (13–15). Thus, evaluating these experimental financing models is greatly limited by the limited historical data of services offered through waivers and other waiver characteristics. Historically, researchers have relied on manual extraction of data from active HCBS waivers in a given year—an unstructured, variable, time-intensive process that may lead to inconsistencies across studies (16–18). Having a uniform method to extract and document these data in order to understand the variation in HCBS waivers is a key precursor to identifying the impacts of HCBS waivers on family-centered care outcomes. Determining a systematic and reproducible approach to characterize Medicaid HCBS waivers is a critical first step to characterize Medicaid HCBS waivers and determine comparative effectiveness.

In this study, we presented a systematic, reproducible approach to capture comprehensive characteristics of Medicaid HCBS waivers. First, we systematically extracted data elements from Medicaid HCBS 1915(c) waivers from 2010 to 2019. Second, we described waiver characteristics over time. This study fills a critical gap in data availability by demonstrating a systematic approach by which researchers can construct a historical, waiver-level database of Medicaid HCBS waiver characteristics.

## Method

### Data Sources

We used current and historical Medicaid 1915(c) waiver applications, renewals, and amendments. States submit waiver applications through an online portal and include a description of the populations to be served, services offered, limitations of services, and the expected number of unique recipients to be served per waiver per fiscal year. We included all waivers providing care to adults aged 65 or older between 2010 and 2019.

First, we downloaded waivers from 2015 to 2019 from the Centers for Medicare & Medicaid Services (CMS) website (as of December 2020). We identified waivers serving adults aged 65 or older by screening the state factsheet provided and downloading relevant waiver documents.

Second, we obtained waiver applications, renewals, and amendments for the years 2010–2014 as collected by Skira et al. (5), who also leveraged the CMS website to identify appropriate waivers.

Third, we incorporated termination dates of waivers as reported by each state through the 372 Form, that is, required reporting for 1915(c) waivers, which we obtained through a Freedom of Information Act request. The 372S form also includes information about expenditures spent on waiver services and the number of people served by each waiver for each year.

### Data Extraction

**Figure 1.**
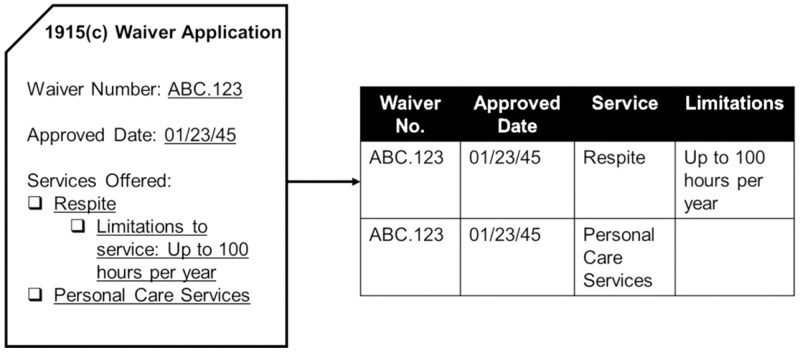
Data extraction example.

Using these waiver documents (*n* = 431), we systematically extracted fields from the Medicaid 1915(c) approved waiver applications for all waivers serving adults ages 65 or older for the years 2010–2019 to construct a database of services provided through waivers by year in each state. Waiver documents exist as either PDF or HTML files. Because HTML files are significantly easier to parse as each text field/table is tagged while PDF files are essentially images, we first converted all waiver applications from PDF files to HTML files by saving PDFs as HTML files in Adobe Acrobat. Then, using the HTML file, we identified the “tag” or “table” corresponding to each data element of interest, for example, the text field that captures the application’s approval date may be tagged as “approval_date.” Then, we used the BeautifulSoup library available in Python to parse the HTML file through a series of commands to extract the content of the desired tag/table and place the content in a cell in an Excel worksheet file. For example, if waiver ABC.123’s approval date was January 1, 2023, then the tag “approval_date” was extracted into a cell of a specified Excel file, see Figure 1. The script repeats this process for all desired data elements. We extracted data elements including waiver approval date, indicator for whether services are provided statewide, types of services provided (eg, personal care services, homemaker, respite), and whether payments may be made to relatives and legally responsible persons (and any associated restrictions).

For quality control, the authors (K.M., E.P., and J.T.) independently assessed all waivers to identify any data incorrectly collected in the data scraping process, conferred over any discrepancies, and, upon consensus of edits, manually updated the database. If data were unable to be scraped from the waiver document, then the authors (J.T. and K.M.) manually extracted data directly from the waiver document to be included in the dataset. See Supplementary Material for the list of waivers extracted manually and the protocol to identify data elements.

### Measures

We categorized a waiver as offering a particular service based on a search for relevant terms in the service name listed in each application. The identification of search terms was informed by (a) the Medicaid HCBS Taxonomy, which categorizes HCBS services provided through Medicaid to allow for consistent identification of services across states, which may use different names for the same services, and (b) a visual inspection of all titles of waiver services (19–22). We examined respite, transport, meals/dietary/nutrition services, caregiver training, and whether payments for personal care or similar services were offered as noted in Appendix C: Participant Services, Section C-1: Summary of Services Covered, Section C-2.a. Waiver Services Summary.

*Respite services* included those provided both in- and out-of-home. We categorized waivers as having respite if “respite” was included in the service name.

*Transportation services* included nonmedical transportation, such as transportation to/from other waiver services, for example, adult day services. We categorized waivers as having transportation if “transport” or “transportation” was included in the service name.

*Meals/dietary/nutrition service* captured services such as, but not limited to, Meals on Wheels, which delivers prepared meals to a waiver recipient’s home and assistance for individuals to improve nutritional intake such as nutritional counseling. We categorized waivers as having meals/dietary/nutrition service if “meal,” “nutrition,” or “dietary” is included in the service name.

*Caregiver training/education/support* varied widely and could include training for an unpaid/family caregiver focused on aiding a care recipient with activities of daily living, medical care, and/or counseling/peer support/family therapy for the caregiver. We categorized waivers as having caregiver training/education/support service if “caregiver,” “caregivers,” “care giver,” “care givers,” “carepartner,” “carepartners,” “care partner,” “care partners,” “caregiving,” “family training” was included in the service name.

*Payments to family caregivers* were identified by examining whether payments to legally responsible individuals and family members for personal care services or other waiver services were allowed in the waiver as defined in (a) Appendix C: Participant Services, Section C-2: General Service Specifications, Section C-2.d. Provision of Personal Care or Similar Services by Legally Responsible Individuals and (b) Appendix C: Participant Services, Section C-2: General Service Specifications, Section C-2.e. Other State Policies Concerning Payment for Waiver Services Furnished by Relatives/Legal Guardians.

### Approach

First, we used the dataset of services available at the waiver-year level and incorporated the termination date of each waiver from the 372 Form.

Second, we described services available through Medicaid 1915(c) waivers regarding the type of service (eg, respite, transportation) and payments to caregivers by state over time. We aggregated all waiver services to the per-waiver per-year level.

We extracted data from waivers using Python and conducted data cleaning, dataset construction, and all analyses in Stata MP (Version 17). This study received Institutional Review Board approval from the authors’ institutions.

## Validation and Results

**Figure 2.**
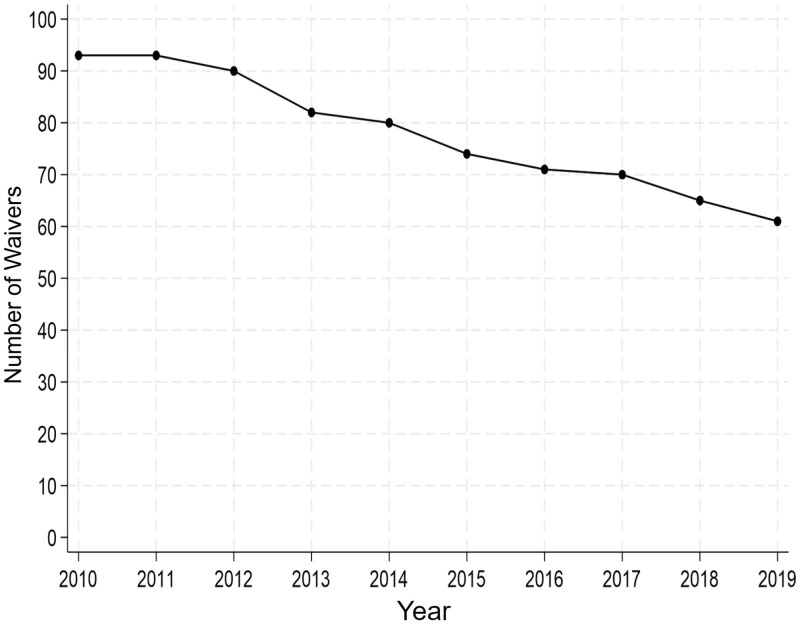
Number of waivers per year serving adults 65+. Source: Author calculations. *Note*: The figure shows the number of active 1915(c) waivers in a given year.

We successfully extracted data systematically using our program from 419 of 431 waiver documents (97%) representing 882 waivers-years across 46 states, effective from 2010 to 2019. During a manual quality control review of data extracted, 9% of waiver documents (*n* = 37) required any manual corrections, with only 4% (*n* = 16) requiring significant corrections impacting analysis, for example, adding in missing services offered by the waiver. We observed that the number of 1915(c) waivers decreased over time, once taking into account the termination dates, as aligned with findings from Skira et al. (5), see Figure 2.

**Figure 3.**
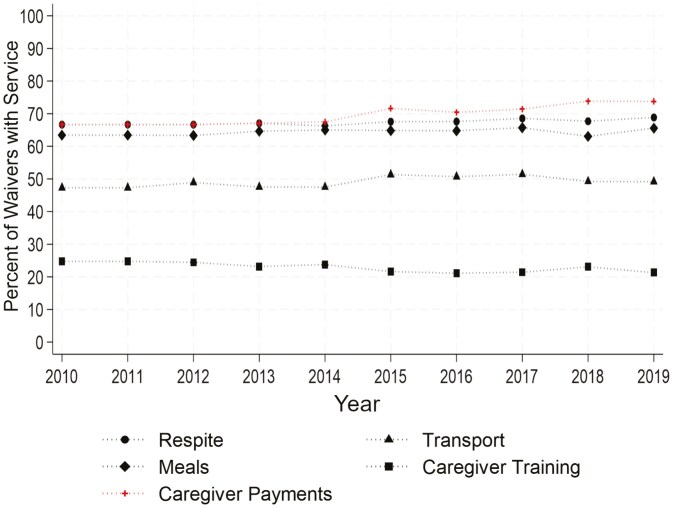
Percent of waivers offering each service per year. Source: Author calculations. *Note*: The figure shows the percentage of active 1915(c) waivers offering each type of service in a given year.

When examining the percentage of waivers offering each type of service (Figure 3), we observed that, since 2010, the percentage of waivers offering each service has largely remained constant, with slight increases in some services. For example, most waivers included respite services, and the percentage of waivers offering respite increased slightly from 66.7% to 68.9%. Similarly, transportation and meal/dietary/nutrition services were often offered. The percentage of waivers offering transportation services increased from 47.3% in 2010 to 49.2% in 2019, while the percentage offering meal/dietary/nutrition services increased from 63.4% to 65.6%. Notably, the percentage of waivers that allow for payments to family caregivers increased from 66.7% in 2010 to 73.4% in 2019, while the percentage of waivers with caregiver training declined from 24.7% to 21.3% over the same time.

## Discussion

We provided proof of concept for a strategy to systematically extract Medicaid 1915(c) waiver characteristics from application documents. We demonstrated how this proof of concept can be combined with state-reported data to CMS to create a novel dataset to describe Medicaid waiver services. We demonstrated that the percentage of waivers offering respite, transportation, meal/dietary/nutrition services increased over time while the percentage of waivers with caregiver training declined. Notably, the percentage of waivers allowing for payments to family caregivers for personal care services and related waiver services increased over time. This work has multiple implications for research focused on Medicaid HCBS as it pilots a methodology to characterize Medicaid HCBS 1915(c) waivers, which can be extended to capture additional 1915(c) components as well as to other Medicaid HCBS waivers (eg, 1915(j), 1915(i)).

Despite the intention of Medicaid 1915(c) waivers to be a mechanism for states to experiment with the delivery of HCBS, 1915(c) waivers have become an established delivery system of HCBS in the United States. The limited historical data of waiver program attributes pose significant challenges to evaluating the effectiveness of these varying models of HCBS as the effects of waiver programs may vary by waiver characteristics (eg, type and availability of services). The data collection approach used in this study to systematically document and describe which services were offered and support for caregivers can help address the documentation challenges historically facing researchers and evaluators. Additionally, the proposed method can capture the number of waiver enrollees expected to be served per waiver per year, which could provide researchers and evaluators with a measure of the expected degree of penetration of services. Our data collection method can further be expanded to capture additional types of services and limitations of services offered through Medicaid 1915(c) waivers within and across states and over time, such as home health, transportation, and personal care assistance. During a quality assurance check, our data collection method yielded 9% of waivers with any error occurring in variables used for analysis. For context, manual chart reviews of electronic health records yielded an overall error rate of 10% (23). Additionally, our findings of the number of states providing training to family caregivers were consistent with other reports conducting manual extraction (24). Given the substantial burden of manually extracting waiver features or reliance on surveys of Medicaid programs (eg, as conducted by KFF), this study’s data collection method is an important first step toward building a systematic record of Medicaid waivers. Future work to refine data extraction to reduce the error rate is a key next step.

Our analysis is subject to limitations. First, we may have an incomplete set of Medicaid 1915(c) waivers for the years 2015–2019 as we relied on data published on the CMS website. Thus, if an amendment or renewal is not available online, it is possible that it was excluded from the analyses. When considering the interpretation of the percentage of waivers offering a service, it is important to note that this could reflect waiver consolidated or incorporation into a state plan or another waiver program(5). Thus, we may have underestimated the availability of waiver services per year. Finally, we use a straightforward search strategy to identify services based on the HCBS taxonomy (20). Future work should include a more in-depth analysis of waiver services descriptions (beyond just service name) to ensure correct classification of all services and consider more granular categories of services to understand potentially differential effects across different services. However, future work to extract the HCBS taxonomy code from services in the waiver applications will allow for more direct mapping of waiver services to other data sources (eg, 372S forms reporting the expenditures per HCBS taxonomy service per waiver per year).

## Conclusion

We provided a methodology to contribute an important piece of the puzzle to better understand the services available through the HCBS 1915(c) Medicaid waivers. Our approach allowed for a deeper understanding of the variation in services across states by systematically extracting data from the waivers themselves. This work lays the foundation for future work to conduct state-by-state analyses of HCBS over time, examine other key aspects of waivers among different target populations, and map waiver services against the Medicaid service taxonomy to facilitate future evaluations. Future work could apply this methodology to applications from more recent years of data to examine how trends in Medicaid HCBS have changed in the wake of the COVID-19 pandemic. Researchers may also use data extracted using this methodology to investigate the effects of these services and inform evidence-based policy supporting community-dwelling older persons with disabilities and their family caregivers.

## Supplementary Material

igaf044_suppl_Supplementary_Materials

## Data Availability

The source data is publicly available through the Centers for Medicare & Medicaid website. Code for data extraction can be made available upon request as original work with the data set is ongoing. The study was not preregistered.
