# Supplementary material for: Systematic Documentation of State Variation in Medicaid Home- and Community-based Services: The Medicaid Home and Community-based Services Dataset Initiative
Source: Innov Aging. 2025 May 6;9(6):igaf044. doi: 10.1093/geroni/igaf044 (PMC12202006; doi:10.1093/geroni/igaf044)
Supplement: igaf044_suppl_Supplementary_Materials [file igaf044_suppl_supplementary_materials.docx]

***Innovation in Aging* Supplementary Material: Miller, Parrish, & Thunell. Systematic Documentation of State Variation in Medicaid Home- and Community-Based Services: The Medicaid Home and Community-Based Services Dataset Initiative.**

**Section 1:**

The files listed below were examined manually by the research team.

- /Elderly and Disabled Medicaid Waivers only/MN/MN_ElderlyWaiver_Dec2011Letter.html
- /Elderly and Disabled Medicaid Waivers only/AL/AL.0068/AL.0068.91.R4.htm
- /Elderly and Disabled Medicaid Waivers only/DC/DC.0334.90.R1.02.htm
- /Elderly and Disabled Medicaid Waivers only/GA/GA.0112.R05.03.htm
- /Elderly and Disabled Medicaid Waivers only/GA/GA.0112.R06.00 (Email).htm
- /Elderly and Disabled Medicaid Waivers only/GA/GA.0112.R06.01 (Email).htm
- /Elderly and Disabled Medicaid Waivers only/KY/KY.0144/KY.0144.R05.00.htm
- /Elderly and Disabled Medicaid Waivers only/KY/KY.0144/KY.0144.R05.01.htm
- /Elderly and Disabled Medicaid Waivers only/KY/KY.0144/KY.0144.R05.02.htm
- /Elderly and Disabled Medicaid Waivers only/MA/MA.0059/MA.0059.R05.00.htm
- /Elderly and Disabled Medicaid Waivers only/MA/MA.0059/MA.0059.R05.01.htm
- /Elderly and Disabled Medicaid Waivers only/MA/MA.0059/MA.0059.R05.02.htm
- /Elderly and Disabled Medicaid Waivers only/OK/OK.0809/OK.0809.R00.00.htm
- /Elderly and Disabled Medicaid Waivers only/VA/VA.40206/VA.40206.R01.00.htm
- /Elderly and Disabled Medicaid Waivers only/WA/WA.0049/WA.0049.R06.02.htm
- /Elderly and Disabled Medicaid Waivers only/WY/WY.0236/WY0236R0500.htm

The authors completed an Excel sheet as follows:

1. Update the document_id with the state abbreviation, a period, then the number of the waiver as labeled in the zipped folder in column A
2. On the first page of the waiver, fill in the proposed effective date and the approved effective date in columns B and C, respectively.
3. Mark whether waiver is renewal, new or replacement as noted on the first page in column E
4. Go to Appendix C-1 by searching “C-1: Summary of Services”.
5. Review Waiver Services Summary list immediately following “C-1: Summary of Services”.
6. Extract services related to caregiver training/education/support and report under “Service” (column D in Excel sheet)
7. Using the title of the service name, search for the service name is section C-1/C-3: Service Specification
8. Under the service specification, then copy and past the text from the text box title “Specify applicable (if any) limits on the amount, frequency, or duration of this service:” in column F; mark whether service delivery method is participant-directed and/or provider managed in column G; and mark whether a legally responsible person, relative and/or legal guardian can provide the service in column H.
9. Sear**c**h for **“Provision of Personal Care or Similar Services by Legally Responsible Individuals” which will appear under section C-2. Indicate whether the state makes payments to legally responsible individuals in column I and copy and paste the text box after this section in column J to capture any limitations/definitions.**
10. Go to the section immediately below, C-2.e. “Other State Policies Concerning Payment for Waiver Services Furnished by Relatives/Legal Guardians”. Copy/Paste text that is marked in column K and then copy/paste the text from the box below the selection into column L.
11. Go to section 4 “Waiver(s) Requested” (at the top of the document). Look at section “C. Statewideness”. Indicate in column M Yes/No whether or not the state requests a waiver of statewideness requirements. If yes, then indicate in column N whether geographic limitation is checked and/or column O if the limitation implementation of participant-direction is checked.
12. Search for “B-3: Number of Individuals Served” (in Appendix B). Under B-3.a. Unduplicated number of participants, insert the unduplicated number of participants from year 1 in column P, year 2 in column Q, year 3 in column R, year 4 in column S and year 5 in column T.

**Section 2:** Supplementary Table 1. Data Elements Extracted Mapped to Waiver Section

| **Data Element** | **Waiver Section** |
| --- | --- |
| Approval Date, Effective Date | 1. Request Information (1 of 3) 1.E. Proposed Effective Date and Approved Effective Date |
| Statewideness | 4. Waiver(s) Requested, 4.C. Statewideness |
| Services (e.g., Personal Care, Respite) | Appendix C: Participant Services, Section C-1: Summary of Services Covered, Section C-2.a. Waiver Services Summary |
| Limitations of Services (e.g., limitations/restrictions on use of respite care) | Appendix C: Participant Services, Section C-1/3: Service Specification |
| Payments to family members and legally responsible individuals | Appendix C: Participant Services, Section C-2: General Service Specifications, Section C-2.d. Provision of Personal Care or Similar Services by Legally Responsible Individuals.  and  Appendix C: Participant Services, Section C-2: General Service Specifications, Section C-2.e. **Other State Policies Concerning Payment for Waiver Services Furnished by Relatives/Legal Guardians.** |
| Number of unique respondents served per waiver per year | Appendix B: Participant Access and Eligibility, B-3: Number of Individuals Served (1 of 4), B-3.a. Unduplicated Number of Participants. |
